# On the Dissection of Living Animals

**Published:** 1816-09

**Authors:** 


### *For the London Medical and Physical Journal.*

### *On the Dissection of Living Animals;* by E. H.

THE laudable and humane manner in which you have set the mark of your disapprobation on the rage of the philosophers of the present day for making experiments, at the expence of so much suffering to the animal creation, does not constitute the least claim of your valuable Journal on the gratitude of the profession. The great object of medical science is the alleviation of human misery; and when any assistance is obtained towards that by experiments made on inferior animals, it cannot be objected to. But this object has been too often lost sight of, and an incalculable degree of protracted suffering has been inconsiderately inflicted, to gratify the vanity of obtaining distinction by the relation of numerous experiments, or to establish hypotheses, which contribute to throw no light on the treatment of disease. It would be illiberal and unjust to assert that we have derived no improvements from this source; but it must be acknowledged that the progress of physiology, the department of medicine most likely to be promoted in this way, has been by no means commensurate with the sacrifice of feeling at which it has been obtained. The researches of Richerand, however creditable to his industry and perseverance, cannot be considered as conferring much honour on his humanity, when he informs us that he has opened more than one hundred animals, during the process of digestion, to ascertain whether the separation of chyle was effected entirely in the duodenum.* The more recent, and, perhaps, not less extensive, experiments of Orfila, in discovering the effects of the various mineral and vegetable poisons, and their respective antidotes, although tending more directly to a practical result, have been unnecessarily profuse. And when it is considered that the effects of poisons are very different in some animals than in the human subject,† it renders it still more reprehensible to repeat such cruelties. How different to the conduct alluded to was that of the illustrious Sir William Jones, who would not even deprive a rare and beautiful bird of Hindoostan of its liberty, in order to gratify his curiosity! Whilst the legislature has wisely and humanely interfered to protect the domestic animals

* Vid. his Elements of Physiology, translated by De Lys, p. 115.

† — Edin. Med. and Surg. Journal, vol. v. p. 255.

from from injurious and cruel treatment, it certainly behoves the dignity of science not unnecessarily to augment their sufferings, by indulging in a line of conduct so extremely censurable.

*London; July* 24.

P.S.—Probably your correspondent, who sent you the Latin translation of Hudibras, has or will inform you that it was turned into Latin verse by Dr. Harmar, Greek Professor at Oxford; and is to be met with in the preface to some of the editions of that work.

---

*For the London Medical and Physical Journal.*

